# A scoping review of the self-reported compassion measurement tools

**DOI:** 10.1186/s12889-023-17178-2

**Published:** 2023-11-24

**Authors:** Hu Jiang, Wenna Wang, Yongxia Mei, Zhixin Zhao, Beilei Lin, Zhenxiang Zhang

**Affiliations:** 1 School of Nursing and Health, Zhengzhou University, Henan, China; 2 Nursing Department, The Third Affiliated Hospital of Zunyi Medical University (The First People’s Hospital of Zunyi), Zunyi, China

**Keywords:** Compassion, Self-compassion, Compassion to others, Compassion from others, Scale, Scoping review

## Abstract

**Background:**

Compassion is closely linked to psychological well-being, and several assessment tools have been developed and studied to assess the level of compassion in different populations and for more precise measurement. There is currently a scarcity of comprehensive knowledge about compassion-related assessment tools, and our research provides an overview of these tools.

**Aims:**

To identify scales used to measure compassion from different flows, and to assess their measurement properties and quality.

**Methods:**

Focusing on compassion assessment tools, the authors conducted a thorough search of 10 Chinese and English databases from their establishment until August 14, 2022. Data extracted included the author, year, country, objectives, target population, as well as the primary evaluation content. Using the COSMIN checklist, the methodological quality and measurement properties of the included studies were appraised. This scoping review was registered with the Open Science Framework and followed the PRISMA-ScR (Preferred Reporting Items for Systematic Reviews and Meta-Analyses Extension for Scoping Reviews) checklist.

**Results:**

There were 15,965 papers searched, and 36 compassion-related measurement tools were identified in this study. None of the 36 studies provided possessed all nine psychometric properties, as outlined by the COSMIN criteria. On the basis of a systematic evaluation of quality, measurement qualities were ranked. The results for internal consistency and content validity were relatively favorable, whereas the results for structural validity were variable and the results for the remaining attributes were either uncertain or negative. A Venn diagram was used to illustrate the overlapping groups of compassion measurement tools based on the three-way flow of compassion. An overview of the reference instrument and theoretical basis for the included studies was provided, and half of them did not contain any theoretical or scale-based evidence.

**Conclusion:**

In this study, 36 compassion-related measuring instruments were identified, and the methodological quality and measurement properties of the included studies were acceptable. The included measurements were consistent with flows of compassion. A further focus of further research should be on developing theories in the compassion domain and developing instruments for measuring compassion that are multidimensional, multi-populations, and culturally relevant.

**Supplementary Information:**

The online version contains supplementary material available at 10.1186/s12889-023-17178-2.

## Introduction

In recent decades, positive psychology has received increasing interest from researchers. The term “positive psychology” is generally defined as the use of psychological theory, research, and intervention techniques to investigate the positive, adaptive, creative, and emotionally fulfilling aspects of human behavior [1]. Positive psychology focuses on human strengths and good emotions, and compassion is acknowledged as a viable and useful study topic in this field. Compassion is a key part of human emotional interactions and it contributes to the mental health of individuals as well as harmonious community coexistence. The intellectual development of compassion has a lengthy history. Compassion emerged from a key component of Buddhist philosophy and Christian traditions [2].

Commonly, compassion is described as the awareness and sensitivity to the experience of pain, as well as the desire to alleviate that suffering [3]. Based on evolutionary theory, Paul Gilbert [4] stated that compassion is a profound awareness of another’s suffering paired with the desire to alleviate it. Compassion, as defined by Gu, et al. [5] and Strauss, et al. [6], consists of five elements: the emotional perception and recognition of the suffering of others and the desire to alleviate it, understanding the universality of suffering, feeling moved by the person suffering and emotionally connecting with their distress, and tolerating uncomfortable feelings so that we remain open and accepting of the person suffering.

Existing research indicates that compassion may be measured in three ways: compassion toward others, compassion from others, and self-compassion [2, 7, 8]. Compassion for others and self-compassion have received greater attention and investigation, but compassion from others is a young and rising field of study.

Compassion for others needs a desire to be helpful, the ability to recognize and respond to distress signals (indicators of suffering), the capacity to tolerate any discomfort sensations that may occur, and the capacity to empathically connect with the suffering of others without judgement [2]. Individuals also require compassion for themselves, particularly during difficult times or when they are suffering. Kristin Neff [7] defined self-compassion as a regulation strategy in which feelings of worry or stress are not avoided in favor of being open and sensitive to one’s own suffering, experiencing feelings of care and kindness to oneself, taking an attitude of understanding and not judging one’s own inadequacies and failures, and acknowledging that one’s own experience is part of the shared human experience. Compassion from others refers to our experience of compassion from those around us, including whether or not we perceive them to be supportive and to possess compassion competencies [2].

There is mounting evidence that compassion is essential for health outcomes. Compassion is connected with mental health and psychological well-being, such as anxiety, sadness, and distress [9, 10]. Self-compassion and fear of compassion have both been shown to influence the relationship between poor sleep quality, psychological discomfort, and mental health [11]. Self-compassion is strongly beneficial to health behaviors [12, 13], low-grade inflammation [14, 15] and the management of certain chronic diseases [16–19]. In the long run, compassion for others and self-compassion predict mental and physical well-being [20, 21]. Trindade, et al. [22] proved that patients with breast cancer were more likely to experience depression symptoms when they fear receiving compassion from others.

Currently, mental health is regarded as a crucial issue in public health which requires immediate attention. According to a review published recently, mindfulness has been analyzed from the perspective of public health, providing theoretical and empirical grounds, techniques, potential implications, and preliminary research and actions to incorporate mindfulness in a broader sense [23]. Compassion and mindfulness have a mutual promotion, shared foundations, complimentary effects, and common goals. Individuals, professionals, organizations, and the society can all benefit from compassion. The contribution of compassion in the field of public health includes promoting social equity, enhancing the quality of healthcare services, facilitating health education and promotion, and supporting community engagement and collaboration [24].

While compassion has had a long history of development, there is no theoretical consensus on the development of compassion scales. Kristin D. Neff [25] developed the concept of self-compassion from the Buddhist philosophy, and created the self-compassion scale, which is thought as the first popular measurement tool related to compassion. Due to differences in population and context, many measurements based on the self-compassion scale were developed. In Gilbert’s theory of compassion, compassion occurs in social interaction, in essence, from the self-to-other, the other-to-self, and the self-to-self, which are referred to as flows of compassion [26]. This means that flows of compassion can coexist and impact one another, and they can also be assessed independently [9]. Paul Gilbert, et al. [27] firstly developed the fears of compassion scales which consists three domains. Recently, the Sussex-Oxford Compassion Measures [28] and the Compassion Motivation and Action Scales (CMAS) [29] were developed that measured self-compassion and compassion for others. Research is needed to validate whether existing compassion-related scales are consistent with the flow of compassion, regardless of the general applicability of these scales across populations.

There is a growing interest in developing patient-reported outcome measures (PROMs) in psychology and psychiatry [30]. Over the past two decades, several compassion measures have been developed tailored to various perspectives and demographics with the aim of improving compassion measurement and assessment. Although most measurement tools are legitimate and useful, there is a lack of systematic knowledge of these tools, and future research will have difficulties in making appropriate choices in this regard. Some studies examined compassion related tools, but they did not provide a comprehensive overview of the issue [6, 31]. In order to remedy these gaps, the purpose of our study was to identify scales used to measure compassion from different flows, so as to systematically assess the measurement properties and the quality of included scales.

## Methods

### Study design

We performed a scoping review consistent the PRISMA-ScR (Preferred Reporting Items for Systematic Reviews and Meta-Analyses Extension for Scoping Reviews) checklist [32]. The protocol was registered on the Open Science Framework (https://osf.io/9hfpg) on August 13, 2022, all materials and data are available at doi: 10.17605/OSF.IO/RUACP.

### Search strategy

Ten Chinese and English databases were included in the search, including the CNKI, Wanfang Database, China Biomedical Literature Database, and VIP Database. The English database included PubMed, Embase, Web of Science, CINAHL, Cochrane Library, and PsycINFO. The retrieval method used a combination of subjects and free words. The retrieval time limit was from January 1, 2003 to August 14, 2022, and the references of the literature were also monitored and retrieved. The retrieval formulas for PubMed, for example, were as follows:

- #1: compassion [MeSH Terms].
- #2: self-compassion [MeSH Terms].
- #3: ((((compassion [Title/Abstract]) OR (self-compassion[Title/Abstract])) OR (“self compassiom“[Title/Abstract])) OR (compassion from others[Title/Abstract])) OR (compassion to others[Title/Abstract])
- #4: #1 OR #2 OR #3
- #5: (((((scale[Title/Abstract]) OR (index[Title/Abstract])) OR (instrument[Title/Abstract])) OR (tool[Title/Abstract])) OR (assessment[Title/Abstract])) OR (measurement[Title/Abstract])
- #6: #4 AND #5

The Mandarin search formulas for the CNKI database are as follows:

- #1: 工具[Subject] OR 量表[Subject] OR评估[Subject].
- #2: 同情[Subject] OR 怜悯[Subject] OR 慈悲 [Subject] OR 自我同情[Subject] OR 自我怜悯[Subject] OR 自我慈悲[Subject] OR 自我关怀[Subject].
- #3: #1 AND #2

### Inclusion and exclusion criteria

Inclusion criteria: (1) the research instrument is a tool for measuring compassion; (2) the research material is an original research paper on the development, update, and use of the instrument; (3) Chinese or English literature; and (4) the instrument is self-reported. Exclusion criteria: (1) Literature that has been published repeatedly; (2) Abstracts; and (3) Articles that are not available in full text.

### Data extraction

**Fig. 1:**
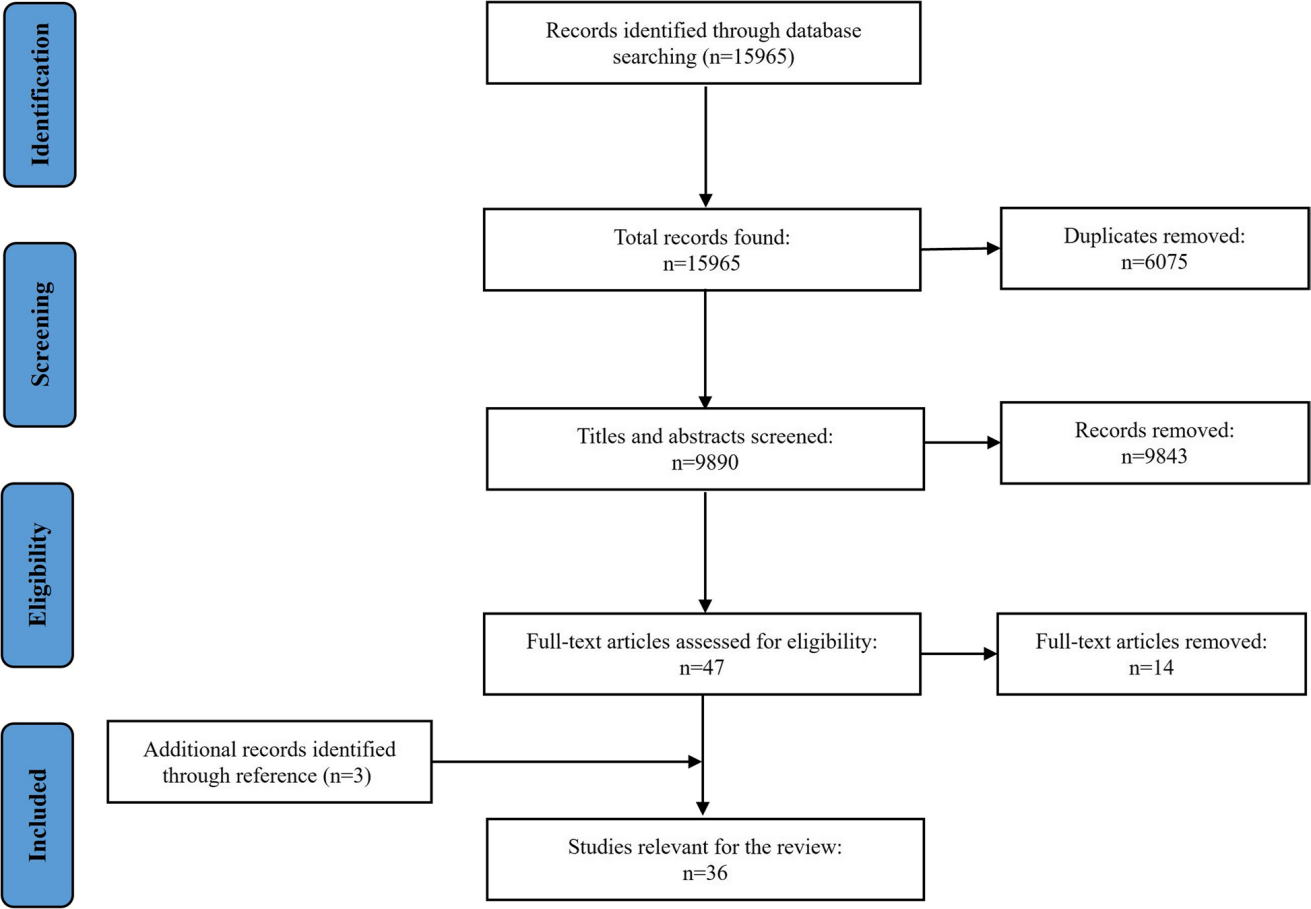
Flow diagram of the screening and selection process of articles identified and excluded

 After the literature review has been conducted. The literatures were imported into EndNote X8 in order to eliminate duplication and manage bibliographies. Two trained researchers independently evaluated the titles and abstracts of the literature for preliminary screening using standardized forms, performed integration and adjustment, and then re-evaluated the literature after reading the complete text. They would discuss and settle any disagreements with the third researcher. The information gathered by various tools consisted of author, year, country, objectives, tool name, target population, and other tool contents. Figure 1 shows the flow of study selection.

### Evaluation of methodological quality and measurement properties of the included studies

Using the (COnsensus-based Standards for the selection of health status Measurement INstruments) COSMIN checklist, methodological quality and measurement properties of the included studies were assessed [33]. The COSMIN checklist includes 9 boxes for classical test theory (CTT) based analyses (internal consistency, reliability, measurement error, content validity, structural validity, hypothesis testing, cross-cultural validity, criterion validity, and responsiveness) to evaluate various design, methodological, and reporting aspects of studies on instruments’ measurement properties. The measurement properties of the identified measures were evaluated in accordance with the criteria for quality of measurement properties defined by Terwee, et al. [34], which may be applied to all nine qualities specified in the COSMIN checklist. Using the COSMIN Risk of Bias Checklist, we assessed the risk of bias [35, 36]. The checklist evaluates the quality of the aforementioned primary outcomes. Each psychometric attribute is evaluated by assigning a score to a series of questions pertaining to its administration and reporting. Each item is assigned a score of “very excellent,“ “adequate,“ “doubtful,“ or “inadequate” in accordance with the COSMIN grading criteria. Depending on the grading of measuring qualities for each research, each property was categorized as positive (+), uncertain (?), or negative (-). The definitions of these quality criteria are provided in the Supplementary Table 1.

## Results

### Study characteristics

After searching ten Chinese and English databases for a total of 15,965 articles and identifying three papers through references, 36 scales were eventually included in this study after literature extraction. The first scale was published by Kristin D. Neff [25], although only three scales were established prior to 2013, the majority of scales were developed in the previous ten years. More than half (19) of the scales came from the United States, 6 from the United Kingdom, and the rest came from Canada, South Korea, Spain, New Zealand, China, and other countries. 13 scales were initially developed and administered to undergraduate students, 6 scales to students and other populations, 4 scales to children and adolescents, 5 scales to patients in clinical practice, 4 scales to healthcare professionals, and 4 scales to others. Table 1 provides an overview of the studies.

**Table 1:** Summary of measurements related to compassion

Author	Country	Scale	Objectives	Target population	Subscales/Dimensions	Total items	Cronbach’s *α*	Self-compassion	Compassion to others	Compassion from others
Zhou, et al. [37]	China	Self-compassionate reactions scale for children (SCRS-C)	To develop an age-appropriate scale to assess children’s self-compassionate reactions by using a vignette technique	Children	6	24	0.81	√	×	×
Sansó, et al. [38]	Spain	Compassionate Leadership Self-reported Scale	To adapt and validate the Compassionate Leadership Self-reported Scale in a sample of palliative care professionals; and to study the relation between compassionate leadership and associated concepts of self-compassion, awareness and self-care	Healthcare professional	4	16	0.723–0.905	×	√	×
K.D. Neff, et al. [39]	USA	The state self-compassion scale (Long- and Short Form)	To create two state measures of self-compassion based on the Self-Compassion Scale	Mechanical Turk workers	6	24	0.944	√	×	×
Sutton, et al. [40]	USA	The self-compassion scale for children (SCS-C)	To assess the reliability and validity of a modified version of the Self-Compassion Scale—Short Form	Children	6	12	0.81–0.83	√	×	×
Paul Gilbert, et al. [2]	UK	The compassionate engagement and action scales	To develop and investigate three new measures of compassion	University students	3	39	0.77–0.94	√	√	√
Sprecher, et al. [41]	USA	Compassionate love scale	To develop a valid and reliable scale to measure compassionate love	Undergraduate students	3	19	0.95	×	√	×
Rose, et al. [42]	Canada	The social self-compassion scale (SSCS)	To describe a domain-specific measure of being self-compassionate in response to interpersonal adversities and challenges	University student, mixed race individuals	2	12	0.74, 0.87	√	×	×
Hwang, et al. [43]	USA	The Santa Clara brief compassion scale (SCBCS)	To develop a brief version of Sprecher and Fehr’s Compassionate Love Scale	Student	1	5	0.90	×	√	×
Paul Gilbert, et al. [27]	UK	Fears of compassion scales	To develop measures of fear of compassion in three domains	Students, therapists	3	45	0.78–0.87	√	√	√
Lown, et al. [44]	USA	The Schwartz Center compassionate care scale™ (SCCCS)	To assess psychometric characteristics of an instrument to measure patient ratings of treating physicians’ compassionate care	Patients	1	12	0.97	×	×	√
Cho, et al. [45]	South Korea	Loving kindness compassion scale (LCS)	To examine the construct of Lovingkindness-Compassion and to development of the Lovingkindness-Compassion Scale	University students	3	15	0.85	×	√	×
Mark Durkin, et al. [46]	UK	The Bolton compassion strengths indicators	To develop and validate a new instrument to measure nurses’ compassion strengths	Undergraduate nursing students	8	48	0.85	×	√	×
Y. Lee, et al. [47]	South Korea	The compassion competence scale	To develop and psychometrically validate the Compassion Competence Scale	Nurses	3	17	0.91	×	√	×
Neto, et al. [48]	Portugal	Short form compassionate love for a partner scale (CLSP-SF)	To develop a short, reliable, and valid instrument to assess compassionate love for a romantic partner	Student	1	5	0.87	×	√	×
Altman, et al. [49]	USA	The body compassion scale	To describe the initial development and validation of the body compassion scale	Undergraduates	3	23	0.92	√	×	×
Kristin D. Neff [25]	USA	The self-compassion scale (SCS)	To define the construct of self-compassion and describe the development of the Self-Compassion Scale	Student	6	26	0.92	√	×	×
Pommier, et al. [50]	USA	The compassion scale (CS)	To develop a measure of compassion for others called the Compassion Scale	Student, community sample	6	16	0.769-0.900	×	√	×
Gu, et al. [28]	UK	The Sussex-Oxford compassion scales (SOCS)	To develop and examine the psychometric properties of new self-report measures of compassion for others and for the self	Health care staff	2	40	0.74–0.94	√	√	×
Steindl, et al. [29]	USA	The compassion motivation and action scales (CMAS)	To develop the Compassion Motivation and Action Scales, and examine its psychometric characteristics.	Adults	3	18	0.879	√	√	×
Oliveira, et al. [51]	Portugal	The compassionate coach scale as perceived by the athlete (CCS-PA)	To develop and validate a measure to assess an athlete’s perception of a coach’s compassionate qualities	Athletes	2	16	0.98	×	×	√
Fernando Iii, et al. [52]	New Zealand	The Barriers to Physician Compassion questionnaire	To describe the development and early psychometric data for a self-report questionnaire assessing barriers to compassion among physicians	Physicians	4	33	0.96	×	√	×
Chang, et al. [53]	USA	The compassion of others’ lives (COOL) scale	To create a survey tool measuring an individual’s level of compassion toward others.	Undergraduate students	2	26	0.98	×	√	×
Catarino, et al. [54]	UK	The submissive compassion scale	To develop a new scale called the submissive compassion scale and compared it to other scales	University students.	1	10	0.89	√	×	×
Crocker, et al. [55]	USA	Friendship compassionate and self-image goals scale	To examine whether relationship goals predict change in social support and trust over time	Undergraduate students	2	13	0.83, 0.90	×	√	×
Falconer, et al. [56]	UK	The self-compassion and self-criticism scales (SCCS)	To develop the Self-Compassion and Self-Criticism Scales, and to examine its psychometric properties	Undergraduate students	2	5	0.87, 0.91	√	×	×
Martins, et al. [57]	USA	The compassion scale	To describe the development of a brief and simple scale for the objective assessment of compassion with specific domains	Undergraduate students and public	1	10	0.82	×	√	×
Burnell, et al. [58]	USA	Compassionate care assessment tool (CCAT)	To explore measures of compassionate nursing care and develop a survey tool	Staff, faculty, and students	4	20	0.774–0.867	×	√	×
Roberts, et al. [59]	USA	The 5-item compassion measure	To develop and validate a tool for measuring patient assessment of clinician compassion	Hospitalized patients	1	5	0.94	×	×	√
S. Sinclair, et al. [60]	Canada	The Sinclair compassion questionnaire (SCQ)	To develop and validate a clinically informed, psychometrically rigorous, patient-reported compassion measure	Patients	1	15	0.96	×	×	√
Raes, et al. [61]	Belgium	The self-compassion scale-short form (SCS-SF)	To construct and validate a short-form version of the Self-Compassion Scale	Patients	6	12	0.87	√	×	×
K. D. Neff, et al. [62]	USA	The self-compassion scale-youth version (SCS-Y)	To develop a comprehensive, age-appropriate, well-validated self-compassion scale for youths	University students	6	17	0.82	√	×	×
Muris, et al. [63]	Netherlands	Shortened self-compassion scale for adolescents (S-SCS-A)	To validate the Shortened Self-Compassion Scale for Adolescents, and to examine relationships among self-compassion, self-esteem, and self-efficacy and symptoms of anxiety disorders and depression	Early adolescents	3	9	0.84	√	×	×
Kemper, et al. [64]	USA	Calm, compassionate care scale (CCCS)	To evaluate two instruments to assess clinician confidence in providing integrative care	Adolescents	1	10	0.95	×	√	×
Zhang, et al. [65]	USA	Single-Item Self-Compassion Scale (SISC)	To develop the Single-Item Self-Compassion Scale (SISC) to measure the self-compassion	Students and community Samples	1	1	NA	√	×	×
M. L. Tanenbaum, et al. [66]	USA	Diabetes-specific Self-Compassion Scale (SCS-D)	To adapt the Self-Compassion Scale and validate it for a diabetes-specific population.	Adults with T1D	2	19	0.94	√	×	×
Molly L. Tanenbaum, et al. [67]	USA	Diabetes-specific self-compassion scale for parents of youth with T1D (SCS-Dp)	To create and assess the psychometric properties of a new tool, the diabetes-specific Self-Compassion Scale	Parents of youth with T1D	2	19	0.94	√	×	×

### Methodological quality and measurement properties

Table 2 summarizes the psychometric features of the 36 studies. None of the 36 studies included all nine psychometric features (internal consistency, reliability, measurement error, content validity, structural validity, hypothesis testing, cross-cultural validity, criterion validity, and responsiveness), according to the COSMIN criteria. Because there was no gold standard instrument of compassion, the criterion validity was not tested. As a result, eight psychometric parameters were assessed. We discovered that one of the included studies didn’t evaluate internal consistency, as indicated by Cronbach’s values. All included studies also reported reliability and content validity. Measurement error was only tested in three studies. There were 33 studies that reported structural validity and 34 studies that reported responsiveness. Four studies reported hypothesis testing. Cross-cultural validity was reported in 9 studies. The measurement properties were assessed based on methodological quality. Internal consistency and content validity were found to be relatively favorable, whereas structural validity was variable, and the remaining attributes were either uncertain or negative. Table 3 displays the measurement properties results.

**Table 2:** The methodological quality of the included studies

Author	Scale	Internal consistence	Reliability	Measurement error	Content validity	Structural validity	Hypothesis testing	Cross-cultural validity	Responsiveness
Zhou, et al. [37]	Self-compassionate reactions scale for children (SCRS-C)	Very good	Doubtful		Very good	Doubtful			Doubtful
Sansó, et al. [38]	Compassionate Leadership Self-reported Scale	Very good	Doubtful		Doubtful	Doubtful			Doubtful
K.D. Neff, et al. [39]	The state self-compassion scale (Long- and Short Form)	Very good	Doubtful		Doubtful	Doubtful		Doubtful	Doubtful
Sutton, et al. [40]	The self-compassion scale for children (SCS-C)	Doubtful	Doubtful		Doubtful	Doubtful			Doubtful
Paul Gilbert, et al. [2]	The compassionate engagement and action scales	Very good	Doubtful		Doubtful	Doubtful		Doubtful	Doubtful
Sprecher, et al. [41]	Compassionate love scale	Doubtful	Doubtful		Doubtful	Doubtful			Doubtful
Rose, et al. [42]	The social self-compassion scale (SSCS)	Doubtful	Doubtful		Doubtful	Doubtful			Doubtful
Hwang, et al. [43]	The Santa Clara brief compassion scale (SCBCS)	Doubtful	Doubtful		Doubtful	Doubtful			Doubtful
Paul Gilbert, et al. [27]	Fears of compassion scales	Very good	Doubtful		Doubtful	Doubtful			Doubtful
Lown, et al. [44]	The Schwartz Center compassionate care scale (SCCCS)	Very good	Doubtful		Doubtful	Doubtful			
Cho, et al. [45]	Loving kindness compassion scale (LCS)	Very good	Doubtful		Doubtful	Doubtful			
Mark Durkin, et al. [46]	The Bolton compassion strengths indicators	Very good	Doubtful		Doubtful	Doubtful			Doubtful
Y. Lee, et al. [47]	The compassion competence scale	Very good	Doubtful		Doubtful	Doubtful			Doubtful
Neto, et al. [48]	Short form compassionate love for a partner scale (CLSP-SF)	Very good	Doubtful		Doubtful	Doubtful		Doubtful	Doubtful
Altman, et al. [49]	The body compassion scale	Very good	Doubtful		Doubtful	Doubtful			Doubtful
Kristin D. Neff [25]	The self-compassion scale (SCS)	Very good	Doubtful		Doubtful	Doubtful	Doubtful	Doubtful	Doubtful
Pommier, et al. [50]	The compassion scale (CS)	Very good	Doubtful	Doubtful	Doubtful	Doubtful		Doubtful	Doubtful
Gu, et al. [28]	The Sussex-Oxford compassion scales (SOCS)	Very good	Doubtful		Doubtful	Doubtful		Doubtful	Doubtful
Steindl, et al. [29]	The compassion motivation and action scales (CMAS)	Very good	Doubtful		Doubtful	Doubtful			Doubtful
Oliveira, et al. [51]	The compassionate coach scale as perceived by the athlete (CCS-PA)	Very good	Doubtful		Doubtful	Doubtful	Adequate	Doubtful	Doubtful
Fernando Iii, et al. [52]	The Barriers to Physician Compassion questionnaire	Very good	Doubtful		Doubtful	Doubtful			Doubtful
Chang, et al. [53]	The compassion of others’ lives (COOL) scale	Very good	Doubtful		Doubtful	Doubtful			Doubtful
Catarino, et al. [54]	The submissive compassion scale	Very good	Doubtful		Doubtful	Doubtful	Adequate		Doubtful
Crocker, et al. [55]	Friendship compassionate and self-image goals scale	Doubtful	Doubtful		Doubtful	Doubtful			Doubtful
Falconer, et al. [56]	The self-compassion and self-criticism scales (SCCS)	Very good	Doubtful		Doubtful	Doubtful			Doubtful
Martins, et al. [57]	The compassion scale	Doubtful	Doubtful		Adequate	Doubtful			Doubtful
Burnell, et al. [58]	Compassionate care assessment tool (CCAT)	Doubtful	Doubtful		Doubtful	Doubtful	Doubtful		Doubtful
Roberts, et al. [59]	The 5-item compassion measure	Very good	Doubtful		Doubtful	Doubtful			Doubtful
S. Sinclair, et al. [60]	The Sinclair compassion questionnaire (SCQ)	Very good	Very good	Adequate	Doubtful	Doubtful			Doubtful
Raes, et al. [61]	The self-compassion scale-short form (SCS-SF)	Very good	Doubtful		Doubtful	Doubtful		Doubtful	Doubtful
K. D. Neff, et al. [62]	The self-compassion scale-youth version (SCS-Y)	Very good	Doubtful	Adequate	Doubtful	Doubtful			Doubtful
Muris, et al. [63]	Shortened self-compassion scale for adolescents (S-SCS-A)	Very good	Doubtful		Doubtful	Doubtful			Doubtful
Kemper, et al. [64]	Calm, compassionate care scale (CCCS)	Very good	Doubtful		Doubtful				Doubtful
Zhang, et al. [65]	Single-Item Self-Compassion Scale (SISC)	NA	Doubtful		Doubtful			Doubtful	Doubtful
M. L. Tanenbaum, et al. [66]	Diabetes-specific Self-Compassion Scale (SCS-D)	Very good	Doubtful		Doubtful	Doubtful			Doubtful
Molly L. Tanenbaum, et al. [67]	Diabetes-specific self-compassion scale for parents of youth with T1D (SCS-Dp)	Very good	Doubtful		Doubtful	Doubtful			Doubtful

**Table 3:** The measurement properties of the included studies

Author	Scale	Internal consistence	Reliability	Measurement error	Content validity	Structural validity	Hypothesis testing	Cross-cultural validity	Responsiveness
Zhou, et al. [37]	Self-compassionate reactions scale for children (SCRS-C)	+	?		+	-			?
Sansó, et al. [38]	Compassionate Leadership Self-reported Scale	+	?		+	-			?
K.D. Neff, et al. [39]	The state self-compassion scale (Long- and Short Form)	+	?		+	-		?	?
Sutton, et al. [40]	The self-compassion scale for children (SCS-C)	+	?		+	-			?
Paul Gilbert, et al. [2]	The compassionate engagement and action scales	+	?		+	+		?	?
Sprecher, et al. [41]	Compassionate love scale	+	-		+	-			?
Rose, et al. [42]	The social self-compassion scale (SSCS)	+	?		+	-			?
Hwang, et al. [43]	The Santa Clara brief compassion scale (SCBCS)	+	-		+	+			?
Paul Gilbert, et al. [27]	Fears of compassion scales	+	?		+	-			?
Lown, et al. [44]	The Schwartz Center compassionate care scale (SCCCS)	+	?		+	-			
Cho, et al. [45]	Loving kindness compassion scale (LCS)	+	?		+	?			
Mark Durkin, et al. [46]	The Bolton compassion strengths indicators	+	?		+	?			?
Y. Lee, et al. [47]	The compassion competence scale	+	-		+	-			?
Neto, et al. [48]	Short form compassionate love for a partner scale (CLSP-SF)	+	-		+	+		+	?
Altman, et al. [49]	The body compassion scale	+	?		+	+			?
Kristin D. Neff [25]	The self-compassion scale (SCS)	+			+	+	+	+	+
Pommier, et al. [50]	The compassion scale (CS)	+	-	?	+	+		+	?
Gu, et al. [28]	The Sussex-Oxford compassion scales (SOCS)	+	-		+	?		+	?
Steindl, et al. [29]	The compassion motivation and action scales (CMAS)	+	-		+	+			?
Oliveira, et al. [51]	The compassionate coach scale as perceived by the athlete (CCS-PA)	+	+		+	?	+	?	+
Fernando Iii, et al. [52]	The Barriers to Physician Compassion questionnaire	+	?		+	+			?
Chang, et al. [53]	The compassion of others’ lives (COOL) scale	+	?		+	?			?
Catarino, et al. [54]	The submissive compassion scale	+	?		+	-	+		?
Crocker, et al. [55]	Friendship compassionate and self-image goals scale	+	?		+				?
Falconer, et al. [56]	The self-compassion and self-criticism scales (SCCS)	+	?		+	-			?
Martins, et al. [57]	The compassion scale	+	-		+	-			?
Burnell, et al. [58]	Compassionate care assessment tool (CCAT)	+	-		+	+	+		+
Roberts, et al. [59]	The 5-item compassion measure	+	?		+	+			?
S. Sinclair, et al. [60]	The Sinclair compassion questionnaire (SCQ)	+	+	?	+	?			?
Raes, et al. [61]	The self-compassion scale-short form (SCS-SF)	+	+		+	?		?	?
K. D. Neff, et al. [62]	The self-compassion scale-youth version (SCS-Y)	+	?	?	+	+			?
Muris, et al. [63]	Shortened self-compassion scale for adolescents (S-SCS-A)	+	?		+	+			?
Kemper, et al. [64]	Confidence in providing calm, compassionate care scale (CCCS)	+	?		+				?
Zhang, et al. [65]	Single-Item Self-Compassion Scale (SISC)	?	?		?			+	?
M. L. Tanenbaum, et al. [66]	Diabetes-specific Self-Compassion Scale (SCS-D)	+	?		+	+			?
Molly L. Tanenbaum, et al. [67]	Diabetes-specific self-compassion scale for parents of youth with T1D (SCS-Dp)	+	?		+	+			?

+ positive, ? uncertain, - negative

### The overlapping clusters of compassion measurement tools

**Fig. 2:**
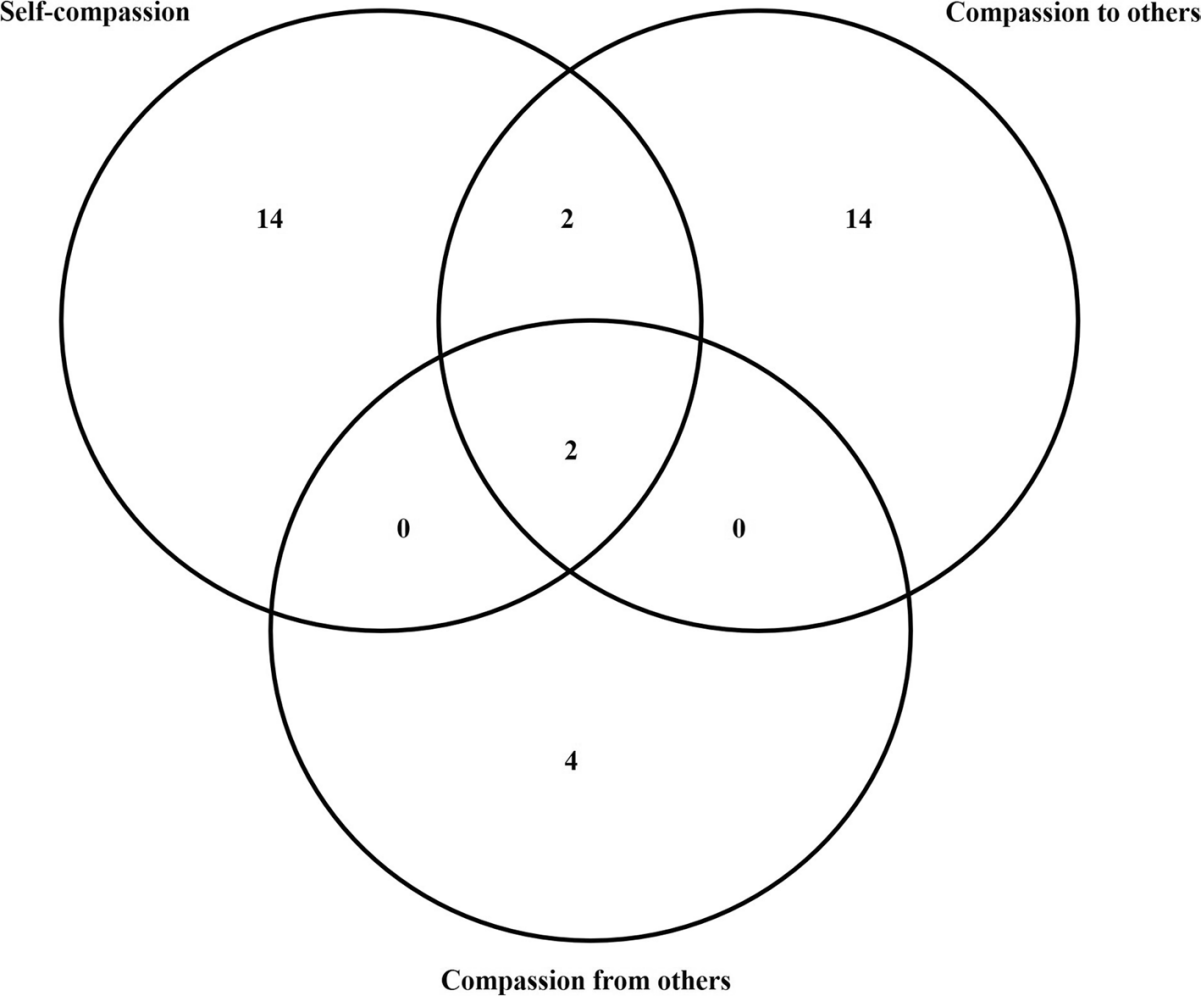
Venn diagram of the overlapping clusters of compassion measurement tools

 A Venn diagram was used to illustrate the overlapping clusters of compassion measurement tools in accordance with the principle of three flows of compassion. Venn diagram is a graphical representation of the intersection and difference relationships between sets. It is made up of two or more sets’ circular areas, with each circular region representing a set and overlapping and non-overlapping regions expressing the intersection and difference between sets. To begin, we created three circles to symbolize the three different flows of compassion, and based on the details of the measurements, we determined the number and relationships of overlapping and nonoverlapping areas, and finalized the Venn diagram with labels. 18 of the 36 measurement tools were related to self-compassion, 18 to compassion toward others, and 6 to compassion from others. Obviously, two scales included all three channels of compassion, while two measures included self-compassion and compassion for others. The results are shown in Fig. 2.

### Reference instrument and theory basis for included studies

Some measurements were developed based on other instruments and theories. Table 4 shows that 19 of the 36 studies included had referenced scales or theoretical underpinnings. More specifically, 15 had references to scales, 6 had theoretical bases, and 2 had both. Despite this, 17 studies did not contain any theoretical or scale-based evidence.

**Table 4:** Reference instrument and theory basis for included studies

Author	Scale	Reference instrument	Theory
K.D. Neff, et al. [39]	The state self-compassion scale (Long- and Short Form)	√	
Sutton, et al. [40]	The self-compassion scale for children (SCS-C)	√	
Paul Gilbert, et al. [2]	The compassionate engagement and action scales		√
Sprecher, et al. [41]	Compassionate love scale	√	√
Rose, et al. [42]	The social self-compassion scale (SSCS)	√	
Hwang, et al. [43]	The Santa Clara brief compassion scale (SSCS)	√	
Paul Gilbert, et al. [27]	Fears of compassion scales		√
Mark Durkin, et al. [46]	The Bolton compassion strengths indicators	√	√
Neto, et al. [48]	Short form compassionate love for a partner scale (CLSP-SF)	√	
Altman, et al. [49]	The body compassion scale	√	
Oliveira, et al. [51]	The compassionate coach scale as perceived by the athlete (CCS-PA)	√	
Fernando Iii, et al. [52]	The Barriers to Physician Compassion questionnaire		√
S. Sinclair, et al. [60]	The Sinclair compassion questionnaire (SCQ)		√
Raes, et al. [61]	The self-compassion scale-short form (SCS-SF)	√	
K. D. Neff, et al. [62]	The self-compassion scale-youth version (SCS-Y)	√	
Muris, et al. [63]	Shortened self-compassion scale for adolescents (S-SCS-A)	√	
Kemper, et al. [64]	Calm, compassionate care scale (CCCS)	√	
M. L. Tanenbaum, et al. [66]	Diabetes-specific Self-Compassion Scale (SCS-D)	√	
Molly L. Tanenbaum, et al. [67]	Diabetes-specific self-compassion scale for parents of youth with T1D (SCS-Dp)	√	

## Discussion

In this study, we used the COSMIN checklist to assess self-reported compassion measurement tools. There were 36 studies found that met the inclusion criteria. The methodological standards of investigations were lowered due to widely ignored assumptions, measurement error, and cross-cultural validity. The majority of studies found instrument results to be acceptable in terms of reliability, content validity, structural validity, and responsiveness. Although fourteen studies referred to other scales, only six had a theoretical basis and were limited by a lack of theory development. Finally, we compiled all 36 measurement tools into a Venn diagram based on compassion’s three-way flow.

Until date, there have been some reviews focused on evaluation of compassion measurement tools [6, 31, 68, 69]. Our research design was a little different from others. We systematically searched and reviewed the literature according to three flows of compassion, and we evaluated all scales according to COSMIN checklist. In addition, the COSMIN checklist served as a tool for critical evaluation of methodological quality and measurement properties. By using the COSMIN checklist to systematically assess and analyze each included study and its corresponding instrument, a summary of the performance of each instrument could be constructed based on a universally accepted standardized framework [70], which was not used in the previous review.

The methodological quality and measurement properties of the included scales were generally acceptable. Except for one article that did not report the Cronbach coefficient, the reported internal consistency ranged from good to excellent (Cronbach’s alpha 0.70) in this review. In terms of reliability, content validity, structural validity, and responsiveness, these measures appear to be valid. The majority of measurements, however, did not report assumptions, measurement error, and cross-cultural validity, which implied that methodological weaknesses existed in these studies, it may affect the reliability and validity of the scales. The applicability of scales to different cultures needs to be strengthened, and more cross-cultural validation of these measures would be a significant advancement. In the majority of cases, these measures retained adequate psychometric properties. In practical terms, it is crucial to seek out and consider various perspectives, so as to ensure the scale is more comprehensive and applicable across different contexts. This approach not only compensates for any shortcomings in the scale’s pre-design but also contributes to its overall effectiveness and validity.

Population and age have led to a diversification of tools for measuring compassion. Children and college students have been the majority of these studies’ target populations to date. Researchers sought to construct universal compassion assessment scales or a single orientation of compassion. The scale was initially designed and validated among college students in order to test one’s compassion level. It was then primarily used to examine self-compassion among teenagers [25]. Several research shown that self-compassion may be acquired from childhood; thus, a number of measures were established for the young population [40, 62]. In clinical practice, patients’ psychological wellbeing is of great concern, caregivers may simultaneously face a variety of stressful and complex situations, the care provided to patients should be compassionate, and caregivers themselves require self-compassion, as measured by scales such as the Compassionate Leadership Self-reported Scale [38], the Bolton Compassion Strengths Indicators [46] and the Sussex-Oxford Compassion Scales (SOCS) [28] were constructed for healthcare professional included medical students and nurses. Sinclair et al. [60] and Roberts et al. [59] both made conjoined attempts to place an emphasis on compassion practice for patients. In addition, compassion measurement tools have been used to athletes and work staffs [39, 51].

It is important to note that our results are consistent with compassion flows in three directions according to Gilbert [2, 8]. Existing compassion scales have predominantly focused on measuring compassion in a single direction, the measurements have evolved based on different perspectives. We discovered that just two scales comprised all three compassion flows, and both were created by Gilbert et al. [2, 27]. In addition, the Sussex-Oxford Compassion Measures (SOCS) [28] and the Compassion Motivation and Action Scales (CMAS) [29] were the only two scales that measured self-compassion and compassion for others. This shift in focus reflects a growing recognition of the importance of understanding and exploring the interplay between different aspects of compassion. Consequently, there is a pressing need to develop multidimensional compassion scales that can capture the complexity and nuances of compassion across various dimensions. This will enable researchers to gain a more comprehensive understanding of compassion and its multifaceted nature. In particular, it explores the interrelationships and internal mechanisms underlying the different compassion flows of individuals, as previous research has indicated that individuals may have inconsistent levels of self-compassion and compassion for others [2].

In the included studies, different methodologies were employed, or developed from various scales. To measure the self-compassion, the Self-Compassion Scale and Short-Form Self-Compassion Scale were most popularly used, and most of self-compassion related scales such as SCS-SF, SCS-Y, S-SCS-A, and SCS-D were derived from them. Further development of research on cultural adjustment and population adjustment in other flows of compassion is necessary to enhance the breadth and depth of studies in this field. Exploring how compassion expresses and adapts across cultures and groups will provide vital insights into the complex dynamics of human compassion, eventually leading to a more inclusive and holistic view of compassion.

To date, there is no consensus about the theory of compassion. Our results showed that the scales developed from different theoretical foundations. Self-compassion was defined by Kristin Neff [7] based on Buddhist perspectives, which is not a theory in the traditional sense. Paul Gilbert [4] developed compassion from evolutionary theory and developed the Compassion Mind model as a result. Moreover, Paul Gilbert, et al. [27] developed fears of compassion scales on the basis of attachment theory. In terms of compassion-related theories, there are relatively few and underdeveloped. M. Durkin, et al. [71] developed the compassion strengths model as the foundation of the Bolton compassion strengths indicators. S. Sinclair, et al. [72] also developed a compassion model to guide the construction of the Sinclair compassion questionnaire. Fernando, et al. [73] did similar work as previous researchers. In light of this, it is evident that there is a dearth of comprehensive theoretical models in the field of compassion. Particularly, the absence of well-established and applicable theories is notable. Therefore, it is imperative to prioritize the development of theories and compassionate assessment tools that are grounded in these theories. Future research should focus on bridging this gap and advancing our understanding of compassion through the development and application of robust theoretical frameworks.

This review has significant implications for future practice and research. From a public health perspective, this study critically examines existing assessment instruments related to compassion and evaluates their quality. The findings of this study can be valuable for psychology researchers, practitioners, educators, and healthcare professionals, providing them with insights and benefits for their respective fields. It is crucial to consider factors such as the target population, age characteristics, and purpose, in order to choose the most suitable compassion assessment tool. In particular, in the context of positive psychology, our study may serve as an inspiration for the design of compassion measurement approaches that can be selected and used by researchers across three different flows, and which can avoid the measurement of a single flow. Our study also offers a theoretical foundation for the development and refinement of scales in future research. Lastly, it is essential to develop compassion scales that are culturally adapted, multidimensional, and encompass a broader range of populations in the future.

### Limitations

Our research has several limitations. To begin, only English and Chinese databases were searched, and non-English and non-Chinese journals were excluded from this study, other potentially relevant literature could have been omitted. In addition, we concentrated on scales that could be used to quantify the three flows of compassion, other measurements such as compassion fatigue and compassion satisfaction were left out of this study, we believe that such a design would allow this study to be more focused. Finally, our analysis focused only on the basic information about the scales rather than exploring in depth their implementation, outcomes, and other indicators, which will limit the generalizability of this study as a result.

## Conclusion

We identified 36 compassion-related measuring instruments, and the included studies’ methodological quality and measurement properties were acceptable. This study also revealed that the included measurements are consistent with three-way compassion flows. Since compassion is such a broad concept, we propose that researchers select appropriate measurement tools based on the needs of measurement and intervention, and that they can also develop more compassion measurement tools suitable for specific populations through further adaptation and validity. Furthermore, future research should concentrate on the development of compassion-related theories to guide and facilitate the application of the scale as well as to promote the field of compassion within a positive psychology perspective.

### Supplementary Information

**Additional file 1. Supplementary Table 1. **Definitions of the measurement properties and their quality criteria.

## Data Availability

All data generated or analyzed during this study are included in this published article and its supplementary information files.

## References

[1] Seligman ME, Csikszentmihalyi M (2000). Positive psychology. An introduction. Am Psychol.

[2] Gilbert P, Catarino F, Duarte C, Matos M, Kolts R, Stubbs J, Ceresatto L, Duarte J, Pinto-Gouveia J, Basran J (2017). The development of compassionate engagement and action scales for self and others. J Compassionate Health Care.

[3] Goetz JL, Keltner D, Simon-Thomas E (2010). Compassion: an evolutionary analysis and empirical review. Psychol Bull.

[4] Gilbert P (2009). The compassionate mind: a new approach to life’s challenges.

[5] Gu J, Cavanagh K, Baer R, Strauss C (2017). An empirical examination of the factor structure of compassion. PLoS ONE.

[6] Strauss C, Lever Taylor B, Gu J, Kuyken W, Baer R, Jones F, Cavanagh K (2016). What is compassion and how can we measure it? A review of definitions and measures. Clin Psychol Rev.

[7] Neff K (2003). Self-Compassion: an alternative conceptualization of a healthy attitude toward oneself. Self and Identity.

[8] Gilbert P (2010). The compassionate mind.

[9] López A, Sanderman R, Ranchor AV, Schroevers MJ (2018). Compassion for others and self-compassion: levels, correlates, and relationship with psychological well-being. Mindfulness.

[10] Whitehead R, Bates G, Elphinstone B, Yang Y (2021). The relative benefits of nonattachment to self and self-compassion for psychological distress and psychological well‐being for those with and without symptoms of depression.. Psychol Psychother: Theory Res Pract.

[11] Kim JJ, Oldham M, Fernando AT, Kirby JN (2021). Compassion mediates poor sleep quality and mental health outcomes. Mindfulness.

[12] Gedik Z (2019). Self-compassion and health-promoting lifestyle behaviors in college students. Psychol Health Med.

[13] Holden CL, Rollins P, Gonzalez M (2021). Does how you treat yourself affect your health? The relationship between health-promoting behaviors and self-compassion among a community sample. J Health Psychol.

[14] Montero-Marin J, Andrés-Rodríguez L, Tops M, Luciano JV, Navarro-Gil M, Feliu-Soler A, López-del-Hoyo Y, Garcia-Campayo J (2019). Effects of attachment-based compassion therapy (ABCT) on brain-derived neurotrophic factor and low-grade inflammation among fibromyalgia patients: a randomized controlled trial. Sci Rep.

[15] Breines JG, Thoma MV, Gianferante D, Hanlin L, Chen X, Rohleder N (2014). Self-compassion as a predictor of interleukin-6 response to acute psychosocial stress. Brain Behav Immun.

[16] Thurston RC, Fritz MM, Chang Y, Barinas Mitchell E, Maki PM (2021). Self-compassion and subclinical cardiovascular disease among midlife women. Health Psychol.

[17] Kılıç A, Hudson J, McCracken LM, Ruparelia R, Fawson S, Hughes LD (2021). A systematic review of the effectiveness of self-compassion-related interventions for individuals with chronic physical health conditions. Behav Ther.

[18] Dowd AJ, Jung ME (2017). Self-compassion directly and indirectly predicts dietary adherence and quality of life among adults with celiac disease. Appetite.

[19] Abdollahi A, Taheri A, Allen KA (2020). Self-compassion moderates the perceived stress and self‐care behaviors link in women with Breast cancer. Psycho‐oncology.

[20] Baer RA, Lykins EL, Peters JR (2012). Mindfulness and self-compassion as predictors of psychological wellbeing in long-term meditators and matched nonmeditators. J Posit Psychol.

[21] Lee EE, Govind T, Ramsey M, Wu TC, Daly R, Liu J, Tu XM, Paulus MP, Thomas ML, Jeste DV (2021). Compassion toward others and self-compassion predict mental and physical well-being: a 5-year longitudinal study of 1090 community-dwelling adults across the lifespan. Transl Psychiatry.

[22] Trindade IA, Ferreira C, Borrego M, Ponte A, Carvalho C, Pinto-Gouveia J (2018). Going beyond social support: fear of receiving compassion from others predicts depression symptoms in Breast cancer patients. J Psychosoc Oncol.

[23] Oman D. Mindfulness for Global Public Health: critical analysis and agenda. Mindfulness. 2023:1–40.

[24] Fahlquist JN (2019). Public health and the virtues of responsibility, compassion and humility. Public Health Ethics.

[25] Neff KD (2003). The development and validation of a scale to measure self-compassion. Self Identity.

[26] Gilbert P (2014). The origins and nature of compassion focused therapy. Br J Clin Psychol.

[27] Gilbert P, McEwan K, Matos M, Rivis A (2011). Fears of compassion: development of three self-report measures. Psychol Psychother: Theory Res Pract.

[28] Gu J, Baer R, Cavanagh K, Kuyken W, Strauss C (2020). Development and Psychometric properties of the Sussex-Oxford Compassion scales (SOCS). Assessment.

[29] Steindl SR, Tellegen CL, Filus A, Seppälä E, Doty JR, Kirby JN (2021). The compassion motivation and action scales: a self-report measure of compassionate and self-compassionate behaviours. Austr Psychol.

[30] Keetharuth AD, Brazier J, Connell J, Bjorner JB, Carlton J, Buck ET, Ricketts T, McKendrick K, Browne J, Croudace T (2018). Recovering quality of life (ReQoL): a new generic self-reported outcome measure for use with people experiencing mental health difficulties. Br J Psychiatry.

[31] Sinclair S, Russell L, Hack T, Kondejewski J, Sawatzky R (2017). Measuring compassion in healthcare: a comprehensive and critical review. Patient.

[32] Tricco AC, Lillie E, Zarin W, O’Brien KK, Colquhoun H, Levac D, Moher D, Peters MDJ, Horsley T, Weeks L (2018). PRISMA extension for scoping reviews (PRISMA-ScR): Checklist and explanation. Ann Intern Med.

[33] Mokkink LB, Terwee CB, Patrick DL, Alonso J, Stratford PW, Knol DL, Bouter LM, de Vet HCW (2010). The COSMIN checklist for assessing the methodological quality of studies on measurement properties of health status measurement instruments: an international Delphi study. Qual Life Res.

[34] Terwee CB, Bot SDM, de Boer MR, van der Windt DAWM, Knol DL, Dekker J, Bouter LM, de Vet HCW (2007). Quality criteria were proposed for measurement properties of health status questionnaires. J Clin Epidemiol.

[35] Prinsen CAC, Mokkink LB, Bouter LM, Alonso J, Patrick DL, de Vet HCW, Terwee CB (2018). COSMIN guideline for systematic reviews of patient-reported outcome measures. Qual life Res: Int J Qual life Aspects Treat care Rehabil.

[36] Mokkink LB, de Vet HCW, Prinsen CAC, Patrick DL, Alonso J, Bouter LM, Terwee CB (2018). COSMIN Risk of Bias checklist for systematic reviews of patient-reported outcome measures. Qual life Res: Int J Qual life Aspects Treat care Rehabil.

[37] Zhou H, Wang YY, Ding JH, Wang Y, Pan JH (2019). Development and validation of an age-appropriate self-compassionate reactions scale for children (SCRS-C). Mindfulness.

[38] Sansó N, Leiva JP, Vidal-Blanco G, Galiana L, West M (2022). The measurement of compassionate leadership: adaptation and Spanish validation of the compassionate leadership self-reported scale. Scand J Caring Sci.

[39] Neff KD, Tóth-Király I, Knox M, Kuchar A, Davidson O (2021). The development and validation of the state self-compassion scale (long-and short form). Mindfulness.

[40] Sutton E, Schonert-Reichl KA, Wu AD, Lawlor MS (2017). Evaluating the reliability and validity of the self-compassion scale short form adapted for children ages 8–12. Child Indic Res.

[41] Sprecher S, Fehr B (2005). Compassionate love for close others and humanity. J Social Person Relat.

[42] Rose AL, Kocovski NL (2021). The Social Self-Compassion Scale (SSCS): Development, validity, and associations with indices of well-being, distress, and social anxiety. Int J Mental Health Addict.

[43] Hwang JY, Plante T, Lackey K (2008). The development of the Santa Clara brief Compassion Scale: an abbreviation of Sprecher and Fehr’s compassionate love scale. Pastoral Psychol.

[44] Lown BA, Muncer SJ, Chadwick R (2015). Can compassionate healthcare be measured? The Schwartz Center compassionate care Scale™. Patient Educ Couns.

[45] Cho H, Noh S, Park S, Ryu S, Misan V, Lee J-S (2018). The development and validation of the lovingkindness-compassion scale. Pers Indiv Differ.

[46] Durkin M, Gurbutt R, Carson J (2020). Development and validation of a new instrument to measure nursing students compassion strengths: the Bolton compassion strengths indicators. Nurse Educ Pract.

[47] Lee Y, Seomun G (2016). Development and validation of an instrument to measure nurses’ compassion competence. Appl Nurs Res: ANR.

[48] Neto J, Neto F (2022). The development of a short form compassionate love for a partner scale. Sex Culture-an Interdiscipl J.

[49] Altman JK, Linfield K, Salmon PG, Beacham AO (2020). The body compassion scale: development and initial validation. J Health Psychol.

[50] Pommier E, Neff KD, Tóth-Király I (2020). The development and validation of the compassion scale. Assessment.

[51] Oliveira S, Rosado A, Cunha M, Ferreira C (2022). The compassionate coach scale as perceived by the athlete: development and initial validation in Portuguese athletes. Int J Sport Exerc Psychol.

[52] Fernando Iii AT, Consedine NS (2014). Development and initial psychometric properties of the barriers to physician compassion questionnaire. Postgrad Med J.

[53] Chang JH, Fresco J, Green B (2014). The development and validation of the compassion of others’ lives scale (the COOL scale). Int J Humanit Social Sci.

[54] Catarino F, Gilbert P, McEwan K, Baiao R, Compassion motivations: distinguishing submissive compassion from genuine compassion and its association with shame (2014). Submissive behavior, depression, anxiety and stress. J Soc Clin Psychol.

[55] Crocker J, Canevello A (2008). Creating and undermining social support in communal relationships: the role of compassionate and self-image goals. J Pers Soc Psychol.

[56] Falconer CJ, King JA, Brewin CR (2015). Demonstrating mood repair with a situation-based measure of self-compassion and self-criticism. Psychol Psychother: Theory Res Pract.

[57] Martins D, Nicholas NA, Shaheen M, Jones L, Norris K (2013). The development and evaluation of a compassion scale. J Health Care Poor Underserved.

[58] Burnell L, Agan DL (2013). Compassionate care: can it be defined and measured? The development of the compassionate care assessment tool. Int J Caring Ences.

[59] Roberts BW, Roberts MB, Yao J, Bosire J, Mazzarelli A, Trzeciak S (2019). Development and validation of a tool to measure patient assessment of clinical compassion. JAMA Netw Open.

[60] Sinclair S, Hack TF, MacInnis CC, Jaggi P, Boss H, McClement S, Sinnarajah A, Thompson G (2021). Development and validation of a patient-reported measure of compassion in healthcare: the Sinclair Compassion Questionnaire (SCQ). BMJ Open.

[61] Raes F, Pommier E, Neff KD, Van Gucht D (2011). Construction and factorial validation of a short form of the self-compassion scale. Clin Psychol Psychother.

[62] Neff KD, Bluth K, Toth-Kiraly I, Davidson O, Knox MC, Williamson Z, Costigan A (2021). Development and validation of the self-compassion scale for youth. J Pers Assess.

[63] Muris P, Meesters C, Pierik A, de Kock B (2016). Good for the self: self-compassion and other self-related constructs in relation to symptoms of anxiety and depression in non-clinical youths. J Child Fam stud.

[64] Kemper KJ, Gascon G, Mahan JD (2015). Two new scales for integrative medical education and research: confidence in providing calm, compassionate care scale (CCCS) and self-efficacy in providing non-drug therapies (SEND) to relieve common symptoms. Eur J Integr Med.

[65] Zhang JW, Howell RT, Chen S, Goold AR, Bilgin B, Chai WJ, Ramis T (2022). I have high self-compassion’: a face-valid single-item self-compassion scale for resource-limited research contexts. Clin Psychol Psychother.

[66] Tanenbaum ML, Adams RN, Gonzalez JS, Hanes SJ, Hood KK (2018). Adapting and validating a measure of diabetes-specific self-compassion. J Diabetes Complicat.

[67] Tanenbaum ML, Adams RN, Wong JJ, Hood KK (2020). Diabetes-specific self-compassion: a new measure for parents of youth with type 1 diabetes. J Pediatr Psychol.

[68] Sinclair S, Kondejewski J, Hack TF, Boss HCD, MacInnis CC (2022). What is the most valid and reliable compassion measure in healthcare? An updated comprehensive and critical review. Patient.

[69] Elices M, Carmona C, Pascual JC, Feliu-Soler A, Martin-Blanco A, Soler J (2017). Compassion and self-compassion: construct and measurement. Mindfulness Compassion.

[70] Li H, Ding N, Zhang Y, Liu Y, Wen D (2017). Assessing medical professionalism: a systematic review of instruments and their measurement properties. PLoS One.

[71] Durkin M, Gurbutt R, Carson J (2019). Stakeholder perspectives of compassion in nursing: the development of the compassion strengths model. J Adv Nurs.

[72] Sinclair S, McClement S, Raffin-Bouchal S, Hack TF, Hagen NA, McConnell S, Chochinov HM (2016). Compassion in health care: an empirical model. J Pain Symptom Manage.

[73] Fernando AT, Consedine NS (2014). Beyond compassion fatigue: the transactional model of physician compassion. J Pain Symptom Manage.

