# Supplementary material for: A scoping review of the self-reported compassion measurement tools
Source: BMC Public Health. 2023 Nov 24;23:2323. doi: 10.1186/s12889-023-17178-2 (PMC10668436; doi:10.1186/s12889-023-17178-2)
Supplement: Supplementary file 1 — Additional file 1. Supplementary Table 1. Definitions of the measurement properties and their quality criteria. [file 12889_2023_17178_MOESM1_ESM.docx]

**Supplementary Table 1** Definitions of the measurement properties and their quality criteria

| **Measurement property** | | | **Description** | **Quality criteria for measurement properties** |
| --- | --- | --- | --- | --- |
| Validity | Content validity | | To which degree the construct assesses whether the items are relevant for the construct to be measured | +: The target population considers all items in the instrument to be relevant AND to be complete  ?: No target population involvement  -: The target population considers the items of the instrument irrelevant OR incomplete |
|  | Construct validity | Structural validity | To which degree the scores of an instrument are an adequate reflection of the dimensionality | +: Factors should explain at least 50% of the variance  ?: Explained variance not mentioned -: Factors explain < 50% of the variance |
|  |  | Hypothesizes testing | To which extent the scores of the instrument are consistent with the theoretically derived hypotheses | +: Correlation with an instrument measuring the same construct ≥ 0.50 or at least 75% of the results are in accordance with the hypotheses AND correlation with related constructs is higher than with unrelated constructs  ?: Solely correlations determined with unrelated constructs  -: Correlations with an instrument measuring the same construct <0.50 OR <75% of the results are in accordance with the hypotheses OR correlation with related constructs is lower than with unrelated constructs |
|  |  | Cross-cultural validity | To which extend the items are an adequate reflection of the original version after translation or culturally adaptation. | +: no important DIF between language versions  ?: DIF not assessed  -: Important DIF found between language versions |
|  | Criterion validity | | To what degree the scores of the instrument are an adequate reflection of a ‘gold standard’. The gold standard should fit the purpose of the  assessed instrument. | +: Convincing arguments that gold standard is “gold” AND correlations with gold standard ≥0.70  ?: No convincing argument that gold standard is “gold” OR doubtful design or method  -: Despite adequate design and method, correlation is < 0.70 |
| Reliability | Reliability | | The proportion of the total variance in the measurements which is  because of ‘’true” differences among patients | +: ICC/weighted kappa ≥ 0.70 OR Pearson’s r ≥ 0.80  ?: Neither ICC/weighted kappa, nor Pearson’s r determined  -: ICC/weighted kappa <0.70 OR Pearson’s r < 0.80 |
|  | Internal consistency | | The extent to which items in a sub(scale) are inter correlated, thus measuring the same construct | +: Cronbach’s α (s) ≥ 0.70  ?: Cronbach’s α not determined  -: Cronbach’s α < 0.70 |
|  | Measurement error | | The systematic and random error of a patient’s score that is not attributed to true changes in the construct to be measured | +:MIC <SDC OR MIC outside the LOA OR convincing arguments that agreement is acceptable  ?: Doubtful design or method OR MIC not defined AND no convincing arguments that agreement is acceptable  -: MIC≥ SDC OR MIC equals or inside LOA, despite adequate design and method |
| Responsiveness |  |  | The ability of the instrument to detect change over time | +: Correlation with an instrument measuring the same construct ≥ 0.50 OR at least 75% of the results  are in accordance with the hypotheses OR AUC ≥ 0.70 AND correlation with related constructs is higher than with unrelated constructs  ?: Solely correlations determined with unrelated constructs  -: Correlation with an instrument measuring the same construct <0.50 OR <75% of the results are in accordance with the hypotheses or AUC <0.70 OR correlation with related constructs is lower than with unrelated constructs. |

DIF Differential item functioning, MIC minimal important change, SDC Smallest detectable change, LOA Limits of agreement, ICC Intra Class Correlation

+= positive rating; ?= indeterminate rating; -= negative rating
